# Big data analysis reveals significant increases in complications, costs, and hospital stay in revision total knee arthroplasty compared to primary TKA

**DOI:** 10.1002/ksa.12499

**Published:** 2024-10-09

**Authors:** Lior Laver, David Maman, Michael T. Hirschmann, Assil Mahamid, Ofek Bar, Yaniv Steinfeld, Yaron Berkovich

**Affiliations:** ^1^ Department of Orthopedics Hillel Yaffe Medical Center Hadera Israel; ^2^ Rappaport Faculty of Medicine Technion University Hospital (Israel Institute of Technology) Haifa Israel; ^3^ Department of Orthopedics Carmel Medical Center Haifa Israel; ^4^ Department of Orthopaedic Surgery and Traumatology Kantonsspital Baselland Liestal Switzerland; ^5^ Faculty of Medicine Lithuanian University of Health Sciences Kaunas Lithuania

**Keywords:** complications, costs, length of stay, mortality, nationwide inpatient sample, R‐TKA, revision total knee arthroplasty, TKA

## Abstract

**Introduction:**

Despite significant advancements in total knee arthroplasty (TKA), some patients require revision surgery (R‐TKA) due to complications such as infection, mechanical loosening, instability, periprosthetic fractures, and persistent pain. This study aimed to explore the specific causes leading to R‐TKA, associated complications, including infection, mechanical failure, and wound issues, as well as costs, mortality rates, and hospital length of stay (LOS) using data from a large national database.

**Methods:**

Data from the nationwide inpatient sample (NIS), the largest publicly available all‐payer inpatient care database in the United States were analysed from 1 January 2016 to 31 December 2019. The study included 44,649 R‐TKA cases, corresponding to 223,240 patients, with exclusions for nonelective admissions. Various statistical analyses were used to assess clinical outcomes, including in‐hospital mortality, postoperative complications, LOS, and hospitalization costs.

**Results:**

Among 2,636,880 TKA patients, 8.4% underwent R‐TKA. R‐TKA patients had higher rates of chronic conditions, including mental disorders (36.4%) and renal disease (9.9%). Additionally, these patients often experienced instability, necessitating revision surgery. Infection (22.3%) was the primary reason for R‐TKA, followed by mechanical loosening (22.9%) and instability. Compared to primary TKA patients, R‐TKA patients exhibited higher in‐hospital mortality (0.085% vs. 0.025%), longer LOS (3.1 vs. 2.28 days), and higher total charges ($97,815 vs. $62,188). Postoperative complications, including blood transfusion (4.6% vs. 1.3%), acute kidney injury (4.4% vs. 1.8%), venous thromboembolism (0.55% vs. 0.29%), infection, and wound problems, were significantly higher in R‐TKA patients.

**Conclusions:**

This study provides detailed insights into t LOS, costs, and complications associated with specific etiologies of revision TKA. Our findings emphasize the need for targeted preoperative optimization and patient education. This approach can help reduce the incidence and burden of R‐TKA, improve patient care, optimize resource allocation, and potentially decrease the overall rates of complications in revision surgeries.

**Level of Evidence:**

Level III.

**Abbreviations**
AKI: acute kidney injury
HCUP: healthcare cost and utilization project
ICD‐10: international classification of diseases, tenth revision
LOS: length of stay
NIS: nationwide inpatient sample
R‐TKA: revision total knee arthroplasty
TKA: total knee arthroplasty
VTE: venous thromboembolism

## INTRODUCTION

Despite significant advancements in total knee arthroplasty (TKA) over the past few decades, many patients still experienced complications such as persistent pain or instability, necessitating revision surgery, known as revision TKA (R‐TKA). Understanding the diverse etiologies leading to R‐TKA, along with the associated complications, costs, mortality rates, and hospital length of stay (LOS), was crucial. This knowledge could optimize patient care, enhance resource allocation within healthcare systems, inform patients about the procedure, and evaluate advancements in TKA that may reduce the need for revision surgeries.

R‐TKA procedures were often more complex and costly than primary TKA, placing a significant financial burden on healthcare systems [10, 17]. Patients undergoing revision surgery faced extended recovery times, increased risks of complications, and potential psychological distress [5, 30]. Revision rates were estimated to range from 1% to 20% [7, 10, 13, 17, 27, 30, 36, 39, 44, 45], highlighting the urgent need for comprehensive analysis of this patient population.

While extensive research had explored various aspects of TKA, the specific characteristics and outcomes associated with R‐TKA were less comprehensively understood. This study addressed this knowledge gap by utilizing a large national database, the nationwide inpatient sample (NIS) [9], to conduct a comparative analysis of R‐TKA and TKA procedures.

The objectives of this study were to analyse the etiologies leading to R‐TKA, assess postoperative complications compared to primary TKA, compare hospitalization costs, evaluate in‐hospital mortality rates, and examine the average hospital LOS for both R‐TKA and primary TKA. By contributing significantly to the existing knowledge base on R‐TKA, this study aimed to improve patient care, enhance resource management, and potentially highlight advancements in TKA procedures that may reduce the need for revision surgeries.

What makes this study original is its use of a large, multiyear data set to identify patterns and specific predictors of R‐TKA that could influence clinical decision‐making, resource allocation, and patient education. The results provide an opportunity to enhance preoperative strategies, reduce the burden of revision surgeries, and potentially lower healthcare costs.

Our hypothesis was that patients undergoing R‐TKA experience significantly higher rates of postoperative complications, longer hospital stays, and increased costs compared to primary TKA patients. The hypothesis also extended to identifying key etiologies such as infection and mechanical loosening as primary drivers for revision surgeries.

## METHODS

This study analyzed data from the NIS, part of the Healthcare Cost and Utilization Project (HCUP) and the largest publicly available all‐payer inpatient care database in the United States. Each dataset entry, termed a ‘case’, represented a group of five patients, matched on clinical parameters. The NIS captures approximately 20% of inpatient stays from HCUP‐associated hospitals, representing about 7 million unweighted admissions annually. The data set used spanned from 1 January 2016 to 31 December 2019. Due to the NIS discharge weight, patient counts were five times larger than case numbers. This extensive data set included 44,649 cases of R‐TKA, corresponding to 223,240 patients.

Nonelective admissions were excluded to focus on planned revision surgeries. We rigorously validated comorbidities and complications through detailed examination of patient‐specific ICD‐10 codes. Analytical techniques, such as annual case visualization, trend identification, and various statistical analyses, were employed. Clinical outcomes examined included in‐hospital mortality, early postoperative complications, LOS, and overall hospitalization costs. Analyses, including cross‐tabulations, one‐way analysis of variance (ANOVA), and independent sample *t*‐tests, adhered to a *p*‐value threshold of less than 0.05 for statistical significance. The Supporting Information S1: Appendix S1 provides an exhaustive list of ICD‐10 procedure codes relevant to this study, including those specific to R‐TKA.

The aetiology for R‐TKA was classified into ten distinct categories, each reflecting unique aspects of complications associated with the procedure: Infection, Cases involving periprosthetic joint infections. Mechanical Loosening, Instances where the prosthetic components loosened, compromising stability. Instability, Situations where the knee joint exhibited abnormal movement or lack of support. Other mechanical complications, mechanical issues not classified as loosening or instability. Pain, persistent pain postprimary TKA, warranting revision. Wear of articular surface, degradation or wear of the prosthetic joint surfaces. Broken prosthesis, fracture or breakage of the prosthetic components. Periprosthetic fracture, fractures occurring around the implant. Arthrofibrosis, dtiffness and limited motion due to excessive scar tissue. Surgical wound complications, issues related to surgical wounds, including poor healing or infection. Other/unspecified cases where the primary ICD‐10 reason did not clearly fit into the above categories or involved less common etiologies. Each category represents a specific reason for revision, enabling a detailed analysis of the different causes and their implications.

A detailed breakdown, including the ICD‐10 codes corresponding to each etiological subgroup, is available in the Supporting Information S1: Appendix S1.

## RESULTS

The total number of patients undergoing TKA during the study period was 2,636,880, with R‐TKA accounting for approximately 8.4% of these cases.

**Figure 1:**
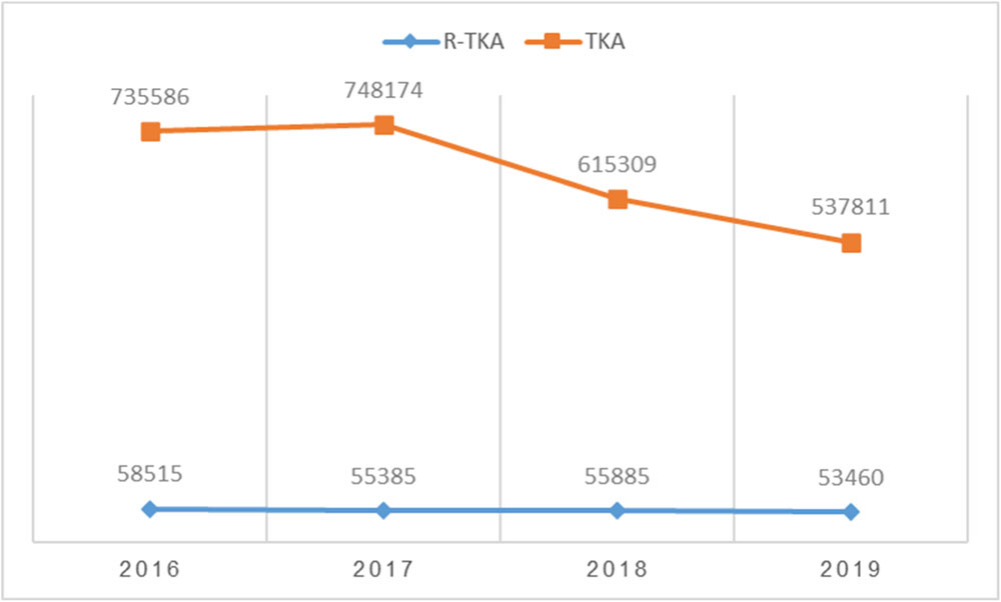
Annual volume of robotic‐assisted total knee arthroplasty (R‐TKA) compared with primary TKA from 2016 to 2019. The graph shows the steady increase in R‐TKA procedures over the years.

**Figure 2:**
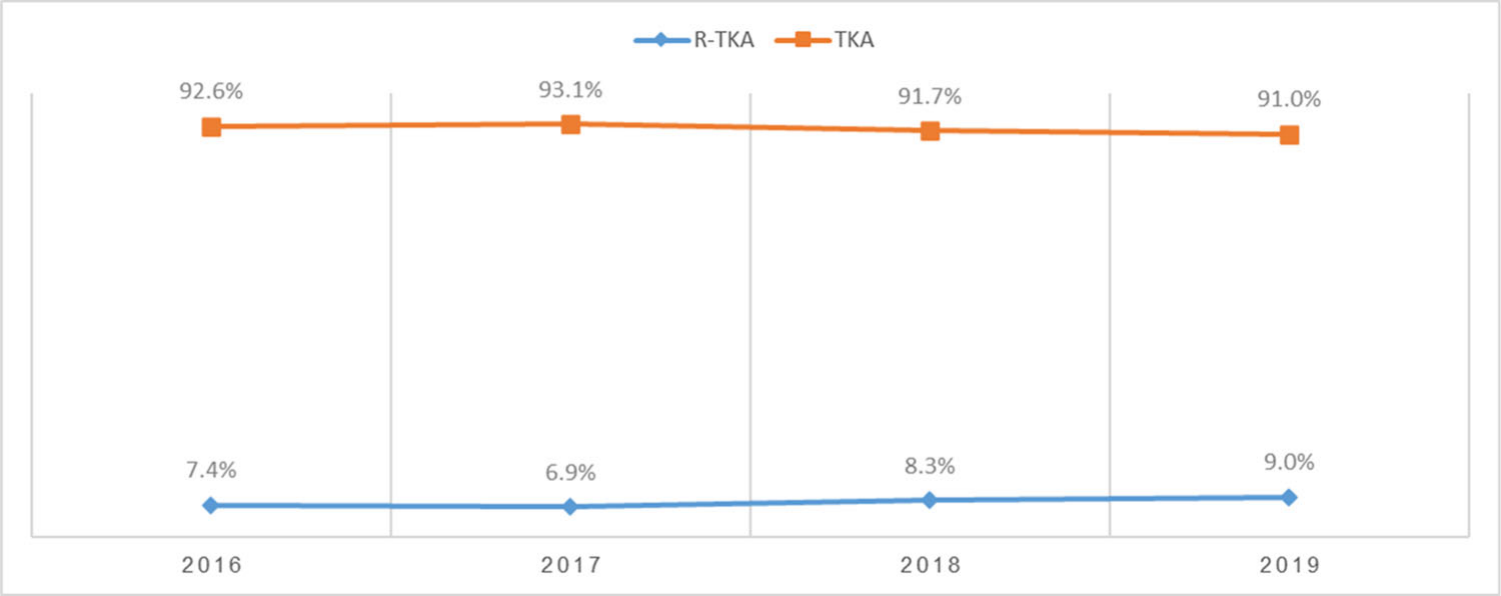
Percentage distribution of annual volume of robotic‐assisted total knee arthroplasty (R‐TKA) compared with primary TKA from 2016 to 2019. The graph highlights the growth in R‐TKA as a proportion of overall TKA procedures.

From 2016 to 2019, 44,649 R‐TKA cases were identified, involving 223,240 patients. Figures 1 and 2 provide an overview of the trends in R‐TKA and primary TKA procedures over the study period. Figure 1 illustrates the annual volume of R‐TKA in comparison to primary TKA, while Figure 2 shows the percentage distribution of the annual volume of R‐TKA compared to primary TKA.

A detailed comparison of demographic and comorbid conditions between R‐TKA and TKA patient groups is provided in Table 1. The percentages (%) represent the prevalence of each condition within the respective group, and significance levels are indicated by *p*‐values. R‐TKA patients demonstrated higher rates of chronic conditions such as mental disorders and renal disease, indicating a more complex medical profile. Conversely, primary TKA patients exhibited higher average age and increased rates of female representation. Furthermore, primary TKA patients presented with a higher prevalence of hypertension and dyslipidemia diagnosis.

**Table 1:** Demographics and comorbidity conditions in robotic‐assisted total knee arthroplasty (R‐TKA) compared with primary TKA.

	R‐TKA (%)	TKA (%)	Significance
Average age (years)	65.34	66.7	*p* < 0.001
Female (%)	56.2	61.6	*p* < 0.001
Obesity	33.2	31.1	*p* < 0.001
Hypertension	57.34	59.4	*p* < 0.001
Dyslipidemia	44.4	46.6	*p* < 0.001
Chronic anaemia	8.1	5.9	*p* < 0.001
Alcohol abuse	1.1	0.9	*p* < 0.001
Osteoporosis	4.3	4	*p* < 0.001
Mental disorders	36.4	29	*p* < 0.001
Parkinson disease	0.75	0.6	*p* < 0.001
Type 2 diabetes	24.8	21.6	*p* < 0.001
Renal disease	9.9	7.1	*p* < 0.001
Congestive heart failure	2.2	1.3	*p* < 0.001
Chronic lung disease	8.8	6	*p* < 0.001

*Note*: Significant differences in age, sex, and various chronic conditions are observed between the two groups, indicating a more complex medical profile in R‐TKA patients.

Table 2 provides a detailed breakdown of the etiological factors contributing to revision‐TKA procedures.

**Table 2:** Aetiology for Revision Total Knee Arthroplasty (R‐TKA) cases.

	Frequency	Per cent
Infection	49,680	22.3
Mechanical loosening	51,175	22.9
Other/unspecified	46,040	20.6
Prosthesis instability	26,845	12.0
Other mechanical complication	21,150	9.5
Pain	16,405	7.3
Wear of articular surface	4725	2.1
Broken prosthesis	2495	1.1
Periprosthetic fracture	1915	0.9
Prosthesis related fibrosis	2005	0.9
Surgical wound complication	810	0.4

*Note*: The table categorizes the primary reasons for revision surgery, with infection and mechanical loosening being the most common etiological factors.

The group ‘other/unspecified’ represents cases where the primary ICD‐10 reason for hospitalization did not explicitly align with the revision, such as those rooted in osteoarthritis of the knee or instances where the etiologies constituted less than 0.1% of revision cases.

A comparison of hospitalization outcomes and costs between R‐TKA and TKA is summarized in Table 3. R‐TKA patients exhibit higher mortality rates during hospitalization, a longer mean LOS, and higher mean total charges compared to primary TKA patients.

**Table 3:** Comparison of hospitalization outcomes and costs between revision total knee arthroplasty (R‐TKA) and primary TKA.

Parameter	R‐TKA (%)	TKA (%)	Significance
Died during hospitalization	0.085%	0.025%	*p* < 0.001
Length of stay mean in days	3.1	2.28	*p* < 0.001
Total charges mean in $	97,815	62,188	*p* < 0.001

*Note*: R‐TKA patients exhibited longer hospital stays, higher mortality rates, and significantly higher total hospital charges compared to primary TKA patients.

Postoperative complications following R‐TKA compared to TKA are presented in Table 4 with statistically significant differences highlighted. Notably, R‐TKA patients had higher rates of blood transfusion, blood loss anaemia, acute kidney injury, heart failure, acute coronary artery disease, pulmonary oedema, venous thromboembolism, and pulmonary embolism, each demonstrating significance when compared to primary TKA patients.

**Table 4:** Comparison of postoperative complications between robotic‐assisted total knee arthroplasty (R‐TKA) and primary TKA.

Parameter	R‐TKA (%)	TKA (%)	Significance
Blood transfusion	4.6	1.3	*p* < 0.001
Blood loss anaemia	22.1	14.8	*p* < 0.001
Acute kidney injury	4.4	1.8	*p* < 0.001
Heart failure	0.2	0.092	*p* < 0.001
Acute coronary artery disease	0.12	0.06	*p* < 0.001
Stroke	0.006	0.007	*p* = 0.783
Pulmonary oedema	0.09	0.06	*p* = 0.003
Venous thromboembolism	0.55	0.29	*p* < 0.001
Pulmonary embolism	0.26	0.2	*p* = 0.029
Pneumonia	0.2	0.2	*p* = 0.148

*Note*: R‐TKA patients experienced higher rates of complications such as blood transfusion, acute kidney injury, and venous thromboembolism compared to TKA patients.

An analysis of the LOS associated with different etiologies in R‐TKA is presented in Table 5. The category ‘surgical wound complication’ required the longest mean LOS, while ‘wear of articular surface’ had the shortest. Significant differences highlight the impact of etiological factors on postoperative recovery time.

**Table 5:** Length of stay associated with different etiologies in revision total knee arthroplasty (R‐TKA).

	Length of stay mean in days	Std. deviation	Significance
Infection	4.76	3.78	*p* < 0.001
Mechanical loosening	2.56	2.08	
Other/unspecified	3.07	3.04	
Prosthesis instability	2.31	1.90	
Other mechanical complication	2.44	2.04	
Pain	2.33	1.92	
Wear of articular surface	2.10	1.39	
Broken prosthesis	2.67	2.79	
Periprosthetic fracture	2.99	2.24	
Prosthesis related fibrosis	2.09	1.22	
Surgical wound complication	4.94	5.93	

*Note*: The table shows significant variations in the mean length of stay depending on the cause of the revision surgery, with surgical wound complications leading to the longest stays.

An analysis of total charges associated with different etiologies in R‐TKA is presented in Table 6. Notably, the etiological category with the highest mean total charges was ‘Periprosthetic Fracture’, while the category with the lowest mean total charges was ‘Wear of Articular Surface’.

**Table 6:** Total hospital charges associated with different etiologies in revision total knee arthroplasty (R‐TKA).

	Total charges mean in $	Std. deviation	Significance
Infection	103,265	125,933	*p* < 0.001
Mechanical loosening	109,935	70,325	
Other/unspecified	98,704	96,137	
Prosthesis instability	83,629	66,983	
Other mechanical complication	95,150	66,071	
Pain	81,907	59,016	
Wear of articular surface	59,618	45,996	
Broken prosthesis	90,201	74,836	
Periprosthetic fracture	117,385	72,587	
Prosthesis‐related fibrosis	73,843	56,835	
Surgical wound complication	69,107	92,138	

*Note*: The table illustrates the significant impact of periprosthetic fractures on total costs, which had the highest mean charges among all etiologies.

Table 7 provides a detailed breakdown of complications associated with different etiologies in R‐TKA. The table presents the percentage occurrence of various complications, ranging from blood transfusion to pneumonia, for each specific etiological category. All the data in the table is statistically significant, except for the occurrence of stroke, which did not exhibit statistical significance difference.

**Table 7:** Complications associated with different etiologies in revision total knee arthroplasty (R‐TKA).

Aetiology/complication	Blood transfusion (%)	Blood loss anaemia (%)	Acute kidney injury (%)	Heart failure (%)	Acute coronary artery disease (%)
Infection	9.6	32.3	8.6	0.3	0.2
Mechanical loosening	2.6	19.4	2.7	0.1	0.1
Other/unspecified	5.6	23.3	4.7	0.3	0.2
Prosthesis instability	1.9	15.3	2.8	0.2	0.1
Other mechanical complication	2.2	17.8	2.5	0.1	0.1
Pain	1.7	16.5	1.8	0.1	0.0
Wear of articular surface	1.3	13.7	2.4	0.1	0.2
Broken prosthesis	3.6	20.2	2.8	0.2	0.0
Periprosthetic fracture	6.0	24.3	4.2	0.5	0.0
Prosthesis‐related fibrosis	1.2	10.5	1.0	0.0	0.0
Surgical wound complication	7.4	21.0	7.4	0.6	0.0

Aetiology/complication	Stroke (%)	Pulmonary oedema (%)	Venous thromboembolism (%)	Pulmonary embolism (%)	Pneumonia (%)
Infection	0.01	0.18	1.0	0.3	0.3
Mechanical loosening	0.01	0.09	0.3	0.2	0.2
Other/unspecified	0.00	0.11	0.7	0.3	0.3
Prosthesis instability	0.02	0.04	0.2	0.2	0.1
Other mechanical complication	0.00	0.05	0.4	0.2	0.1
Pain	0.00	0.00	0.2	0.1	0.2
Wear of articular surface	0.00	0.00	0.2	0.2	0.2
Broken prosthesis	0.00	0.00	0.4	0.2	0.2
Periprosthetic fracture	0.00	0.00	0.0	0.3	0.0
Prosthesis‐related fibrosis	0.00	0.00	0.5	0.0	0.0
Surgical wound complication	0.00	0.00	0.0	1.2	0.0

*Note*: The table presents the prevalence of various complications, such as blood transfusion and acute kidney injury, across different revision etiologies, showing statistically significant differences between the groups.

## DISCUSSION

The most important finding of the present study was the heightened occurrence of early postoperative complications and extended hospitalization duration following R‐TKA compared to primary TKA [18, 26, 31, 34, 42]. Specifically, our analysis revealed that infection was the primary reason for R‐TKA in 22.3% of cases, closely followed by mechanical loosening at 22.9%. Previous literature demonstrated infection and mechanical loosening as the main causes of R‐TKA. In addition, a history of previous surgeries affected the likelihood of TKA revision [4, 6, 12, 16, 17, 24, 32, 33], emphasizing the critical importance of precise surgical procedures and vigilant postoperative monitoring of patients. Furthermore, we observed that R‐TKA was associated with elevated mortality rates, prolonged LOS, and increased mean total hospitalization charges compared to primary TKA. R‐TKA due to joint infection resulted in more anaemia, blood transfusions, AKI, and VTE [8, 28, 37, 41]. R‐TKA due to peri‐prosthetic joint infection and surgical wound complications resulted in the longest LOS. R‐TKA due to periprosthetic fracture, mechanical loosening, and infection resulted in higher total charges.

These findings correlate with previous evidence that R‐TKA performed due to peri‐prosthetic joint infection carried an elevated risk of short‐term morbidity and mortality. Patients who underwent R‐TKA due to infection or periprosthetic fracture faced a notably higher mortality risk compared to the general population, with the risk increasing over time. Conversely, those undergoing R‐TKA due to loosening and/or implant wear had mortality rates similar to those of the general population. Initially, patients with aseptic loosening and/or wear and instability experienced lower mortality rates. Still, over time, a shift to excess mortality occurred, particularly beyond 5 years for instability patients and beyond 10 years for aseptic loosening and/or wear patients [1, 2, 23, 35, 38, 40, 46]. The current study revealed that patients who underwent R‐TKA had more chronic anaemia, type 2 DM, renal diseases, chronic heart failure, and chronic lung failure. These factors may further elucidate the elevated morbidity rates observed in this study.

Previous studies reported an average hospital charge of $71,872 for R‐TKA, compared to $97,815 in our study [3, 11]. This increase in costs is likely due to more recent data reflecting higher healthcare costs and more complex cases being included. Additionally, our study noted a shorter average LOS of 3.1 days for R‐TKA patients compared to 4.44 days in the previous study, which may indicate improvements in surgical techniques and postoperative care over time. Both studies found that hypertension and obesity were the most prevalent comorbidities in R‐TKA patients, highlighting the importance of comprehensive preoperative optimization to reduce the risk of complications. In our cohort, hypertension was present in 57.34% of R‐TKA patients and obesity in 33.2%, compared to the mentioned study's findings of 65.04% and 22.98%, respectively. These findings collectively emphasize the need for enhanced preoperative planning, patient selection, and perioperative optimization to mitigate the clinical and financial burdens of revision surgeries [25]. The projected increase in TKA and R‐TKA volumes underscores the importance of efficient resource utilization and improved perioperative management to enhance patient outcomes and reduce healthcare costs.

Though often necessary, R‐TKA surgery raises a multifaceted discussion encompassing various significant factors. Reviewing the literature, it has been shown that the leading causes prompting revision are infection and mechanical loosening [14, 15, 36, 43], highlighting the importance of meticulous surgical technique and ongoing patient monitoring. The decision to revise a TKA also has substantial financial implications. Due to increased complexity and extended recovery periods, the costs of R‐TKA are notably higher than those of primary TKA. The overall average cost of R‐TKA surpasses that of TKA by approximately 35,000 USD. This disparity cannot solely be attributed to the higher prevalence of comorbidities in R‐TKA patients; it is also influenced by the documented higher costs associated with septic replacements [29]. It is important to note that the current analysis included only two‐stage revision surgeries. Choosing between a one‐ or two‐stage procedure can directly impact the expenses. A recent study conducted in Australia demonstrated that the preferred approach for patients with periprosthetic joint infection who do not necessitate a two‐stage exchange arthroplasty is to adopt a one‐stage R‐TKA. A one‐stage exchange should be recommended for eligible patients who meet the criteria [30].

This study has several limitations. Although the NIS offers extensive healthcare data on large populations, potential errors in the database may arise from suboptimal coding and manual data entry [3]. Furthermore, the NIS only captures data during the in‐hospital period [19, 20, 21, 22], lacking long‐term follow‐up input and postoperative outcomes. Despite these limitations, the NIS database has proven invaluable for observational epidemiological studies and has been validated as a reliable source of comorbidity and complication data. Additionally, physicians’ choice of surgical intervention could introduce a selection bias into our study, as evidenced by differences in comorbidities between the R‐TKA and primary TKA groups in the initial database.

Another limitation stems from the fact that the NIS database does not provide detailed information on factors, such as surgery length, type of anaesthesia, and tourniquet time, which could impact the immediate postoperative period. However, it is also one of the benefits of using the NIS database, as it portrays the gross and true landscape and impact of a certain procedure on the health system throughout an entire country—not only in the top‐ranked centers with the most experienced surgeons and staff but also taking into account the influence of less experienced surgeons and staff and lower‐volume medical centers on a certain health system.

The current study boasts several strengths worth highlighting. First, it draws upon the most recent and comprehensive data set available in the United States, spanning four consecutive years. This extensive data pool, derived from the largest publicly accessible all‐payer inpatient care database, offers an updated and more robust and representative sample than previous studies. This research thoroughly explores these surgical interventions by examining demographics, health comorbidities, early postoperative clinical outcomes, and the ensuing economic burden associated with R‐TKA and TKA. Second, the study focuses specifically on the often‐neglected early postoperative period, which, despite its significance, is frequently overshadowed by long‐term outcomes. Insights gained during this critical phase can potentially mitigate future complications and enhance clinical and economic outcomes over the long term. Lastly, incorporating the ICD10 coding system ensures the study's relevance and applicability to contemporary medical coding practices.

## CONCLUSIONS

Revision TKA was associated with elevated mortality rates, prolonged LOS, and increased mean total hospitalization charges compared to primary TKA. Prosthetic joint infection was the primary reason for R‐TKA in the United States, closely followed by mechanical loosening. R‐TKA due to joint infection may have been associated with more postoperative anaemia, blood transfusions, AKI, and VTE. R‐TKA due to peri‐prosthetic joint infection and surgical wound complications may have resulted in longer LOS. R‐TKA due to periprosthetic fracture, mechanical loosening, and infection may have been associated with higher total charges.

Identifying specific risks and financial implications associated with various causes of revision TKA could aid in improving and optimizing targeted pre‐ and postoperative care, as well as patient education. This could help reduce the burden of R‐TKA. Enhanced understanding of these factors can improve patient care, optimize resource allocation, and potentially reduce complications of R‐TKA surgeries.

## AUTHOR CONTRIBUTIONS

Lior Laver conducted major parts of the work, manuscript writing. David Maman conducted major parts of the work, data analysis, and manuscript writing. Michael T. Hirschmann provided clinical expertise. Assil Mahamid contributed to manuscript writing. Ofek Bar contributed to manuscript writing. Yaniv Steinfeld contributed to revision writing Yaron Berkovich conceived the study idea and mentored the project.

## CONFLICT OF INTEREST STATEMENT

The authors declare no conflict of interest.

## ETHICS STATEMENT

The authors have nothing to report.

## Supporting information

Supporting information.

## Data Availability

The data that support the findings of this study are available from the national inpatient sample (NIS). However, restrictions apply to the availability of these data, which were used under license for the current study, and so are not publicly available. Data are available from the authors upon reasonable request and with permission of the NIS.
